# Comparison of salivary carcino embryonic antigen among tobacco users without lesions, oral potentially malignant disorders and oral squamous cell carcinoma

**DOI:** 10.6026/973206300213239

**Published:** 2025-09-30

**Authors:** Padmanathan Renganathan, Harsha Vardhan B.G., Srividhya Srinivasan, Saraswathi Gopal K.

**Affiliations:** 1 Department of Oral Medicine and Radiology, Meenakshi Ammal Dental College, Maher University, Chennai, India

**Keywords:** Carcino embroyogenic antigen, oral cancer, oral potentially malignant disorder

## Abstract

Oral cancer, mainly oral squamous cell carcinoma (OSCC), is a common head and neck malignancy affecting multiple oral sites. This study compared salivary carcinoembryonic antigen (CEA) levels among tobacco users without lesions, patients with oral potentially malignant disorders (OPMDs), and OSCC patients. Thirty subjects were divided into three groups of ten, and unstimulated saliva samples were collected and analyzed using a Can Ag Diagnostics ELISA kit. Data analysis was performed using SPSS v26, with means compared by one-way ANOVA and t-tests. Results showed CEA levels were highest in OSCC, followed by OPMDs and tobacco users, suggesting its role as a non-invasive biomarker for early malignant transformation.

## Background:

Oral cancer is a major global health issue, commonly involving the lips, tongue, buccal mucosa, gingiva, floor of the mouth and oropharynx and accounts for a large share of head and neck cancers [1]. About 90% are squamous cell carcinomas (OSCC). High incidence in India and Asia is mainly due to widespread tobacco and betel quid use. The rising incidence in younger people is linked to tobacco abuse, with men more often affected than women (M:F = 1.5:1). Oral potentially malignant disorders (OPMDs) such as leukoplakia, erythroleukoplakia, oral lichen planus (OLP) and oral submucous fibrosis (OSMF) increase the risk of OSCC [2, 3]. Leukoplakia, the most common, is linked to tobacco [4]. OSMF, due to areca nut chewing, causes fibrosis, trismus and restricted mouth opening, mainly in India and Southeast Asia. OLP is immune-mediated, with a prevalence of 0.5-2.2% [5]. Patients with OPMDs need close monitoring to detect malignant transformation [6]. Despite advances, the 5-year survival rate remains around 50%, with many cases arising from precursor lesions [7]. Delayed detection is often due to the absence of rapid, simple screening methods and symptoms mimicking benign conditions [8, 9]. Affordable, sensitive and portable biosensors using easily collected fluids (saliva, sputum, urine) are needed for early detection [10]. Saliva is a practical diagnostic medium because collection is safe, simple and non-invasive [11]. Measuring salivary CEA may help in early detection, monitoring and treatment assessment [12]. Therefore, it is of interest to evaluate and compares salivary CEA levels in tobacco users, patients with OPMDs and oral cancer cases to determine its diagnostic value and specificity as a salivary biomarker.

## Methodology:

[1] Clinical diagnosis was confirmed by the principal investigator and two senior faculty members.

[2] Detailed habit and disease history has been recorded; patients also received counselling.

[3] The study protocol was explained and informed consent obtained.

## Age groups:

[1] Group A: 20-35 years

[2] Group B: 36-50 years

[3] Group C: 51-70 years

## Sample collection:

Participants will rinse their mouth and abstain from food or drink for at least one hour before saliva collection under unstimulated conditions. 1-2 ml of saliva will be collected before any treatment, centrifuged to remove debris and supernatants stored at-80°C.

## CEA estimation:

CEA levels will be measured using a CanAg Diagnostics ELISA kit. Reagents and samples will be added to ELISA wells, followed by 100 µl of 3,3,5,5-tetramethylbenzidine-HRP substrate. After incubation at 37°C in the dark for 30 min, absorbance will be read at 620 nm using a microplate spectrophotometer per the manufacturer's protocol. The kit detection range is 0.5-50 ng/ml and results will be reported as ng/ml of saliva [1].

## Results:

Data were entered in Microsoft Excel and analysed using SPSS version 26 (IBM, Chicago, USA). Continuous variables were presented as mean ± standard deviation (SD); categorical variables as number and percentage. One-way ANOVA and independent t-tests were used for comparisons. A p-value < 0.05 was considered statistically significant. The mean salivary CEA levels were 391.19 ± 45.67 ng/ml in OSCC patients, 316.13 ± 16.26 ng/ml in patients with premalignant disorders and 242.20 ± 33.74 ng/ml in tobacco users. The differences among the groups were statistically significant (p < 0.001) (Table 1 - see PDF, Figure 1 - see PDF).

## Discussion:

Oral cancer remains a major global health burden with rising incidence and mortality, especially among males due to high-risk habits like tobacco chewing and smoking. Prolonged exposure leads to mucosal changes, progressing from potentially malignant disorders (OPMDs) to oral squamous cell carcinoma (OSCC) [13]. Early detection is vital to reduce morbidity and improve outcomes. While clinical and histopathological diagnoses are standard, advances in molecular biomarkers offer improved precision for screening and monitoring OSCC. Carcinoembryonic antigen (CEA), first described by Phil Gold and Samuel Freedman in the 1960s, is one of the earliest tumor markers identified, playing a role in cell adhesion, invasion and metastasis. Elevated CEA is found in many cancers and certain non-malignant conditions and higher levels are also seen in smokers. This study assessed salivary CEA levels in OSCC patients, OPMD patients and tobacco users without lesions. Results showed a significant stepwise increase in CEA from tobacco users to OPMD cases to OSCC patients, supporting its value as a marker reflecting progression from risk exposure to malignancy. Few studies have compared salivary CEA across healthy users, OPMDs and OSCC in one cohort, positioning this study as important evidence for salivary CEA's role as a non-invasive continuum biomarker for early detection and monitoring in high-risk groups.

## Conclusion:

We show that salivary CEA levels are significantly higher in OSCC patients than in OPMD patients and tobacco users. Among OPMDs, conditions like OSMF and leukoplakia showed higher CEA levels, indicating greater malignant potential. Salivary CEA shows promise as a simple, non-invasive biomarker for early detection and monitoring of oral cancer risk.

## Future scope:

To validate salivary CEA as a standard biomarker for OSCC, large multicentric and longitudinal studies are needed to track OPMD progression and correlate CEA changes. Combining salivary CEA with other markers (e.g., CYFRA 21-1, CA 125, IL-6) could improve diagnostic accuracy. Developing portable point-of-care tests would expand screening access, especially in rural areas. Monitoring salivary CEA before and after treatment could help assess therapy response and prognosis. Future work should also address potential confounders such as infections, inflammation, systemic diseases and smoking cessation to refine its clinical reliability.
